# A naturally occurring membrane-anchored Gα_s_ variant, XLα_s_, activates phospholipase Cβ4

**DOI:** 10.1016/j.jbc.2022.102134

**Published:** 2022-06-13

**Authors:** Hoa T.N. Phan, Joseph Loomis, Saji Abraham, Qing He, Murat Bastepe, Alan V. Smrcka

**Affiliations:** 1 Department of Pharmacology, University of Michigan, Ann Arbor, Michigan, USA; 2 Chemical Biology Program, University of Michigan, Ann Arbor, Michigan, USA; 3 Endocrine Unit, Department of Medicine, Massachusetts General Hospital and Harvard Medical School, Boston, Massachusetts, USA

**Keywords:** phospholipase C, G protein, G protein–coupled receptor, inositol phosphate, signal transduction, Gαs, adenylate cyclase, plasma membrane targeting, AC, adenylate cyclase, BSA, bovine serum albumin, cDNA, complementary DNA, CV, column volume, DMEM, Dulbecco's modified Eagle's medium, Epac, exchange protein directly activated by cAMP, FBS, fetal bovine serum, Gα_s_, G protein αs subunit, GNAS, Guanine Nucelotide binding protein, Alpha Stimulating activity polypeptide, HEK293, human embryonic kidney 293 cell line, IP, inositol phosphate, IP_3_, inositol 1,4,5-trisphosphate, Iso, isoproterenol, β-ME, β-mercaptoethanol, PI, phosphatidylinositol, PLCβ, phospholipase Cβ, PM, plasma membrane, PRR, proline-rich region, PTH, parathyroid hormone, TBS-T, Tris-buffered saline buffer supplemented with Tween-20, XLα_s_, extra-large stimulatory Gα

## Abstract

Extra-large stimulatory Gα (XLα_s_) is a large variant of G protein α_s_ subunit (Gα_s_) that uses an alternative promoter and thus differs from Gα_s_ at the first exon. XLα_s_ activation by G protein–coupled receptors mediates cAMP generation, similarly to Gα_s_; however, Gα_s_ and XLα_s_ have been shown to have distinct cellular and physiological functions. For example, previous work suggests that XLα_s_ can stimulate inositol phosphate production in renal proximal tubules and thereby regulate serum phosphate levels. In this study, we show that XLα_s_ directly and specifically stimulates a specific isoform of phospholipase Cβ (PLCβ), PLCβ4, both in transfected cells and with purified protein components. We demonstrate that neither the ability of XLα_s_ to activate cAMP generation nor the canonical G protein switch II regions are required for PLCβ stimulation. Furthermore, this activation is nucleotide independent but is inhibited by Gβγ, suggesting a mechanism of activation that relies on Gβγ subunit dissociation. Surprisingly, our results indicate that enhanced membrane targeting of XLα_s_ relative to Gα_s_ confers the ability to activate PLCβ4. We also show that PLCβ4 is required for isoproterenol-induced inositol phosphate accumulation in osteocyte-like Ocy454 cells. Taken together, we demonstrate a novel mechanism for activation of phosphoinositide turnover downstream of G_s_-coupled receptors that may have a critical role in endocrine physiology.

G protein–coupled receptors convert signals from the extracellular environment to physiological responses by activating heterotrimeric G proteins. Among four G protein subtypes, Gα_s_, in the GTP-bound form, stimulates adenylyl cyclase to produce cAMP, a second messenger that activates PKA (1), cAMP-regulated guanine nucleotide exchange factors (or Epac [exchange protein directly activated by cAMP]) (2), and cAMP-gated ion channels (3). While Gα_s_ is expressed ubiquitously, its longer and lesser known variant, extra-large stimulatory Gα (XLα_s_) is selectively and abundantly expressed in brain and neuroendocrine tissues throughout development and in the adult (4) with reduced expression in some additional tissues postnatally (5). Loss of XLα_s_ is associated with perinatal growth restriction and feeding difficulties in humans and mice (5).

XLα_s_ is a G protein α-subunit largely identical to Gα_s_ except that the N-terminal α-helix is replaced with an extended N-terminal domain. While Gα_s_ starts at exon 1 at the GNAS (Guanine Nucelotide binding protein, Alpha Stimulating activity polypeptide) complex locus, XLα_s_ is derived from a different upstream promoter, and its first exon splices onto exon 2 (6) (Fig. 1*A*). Activation of XLα_s_ by parathyroid hormone (PTH) results in sustained cAMP production at the plasma membrane (PM) (7). Biochemically, XLα_s_ can form a heterotrimer with Gβγ *in vitro* and can activate adenylyl cyclase in cells (8). In an overexpression setting, XLα_s_ can mediate β2 adrenergic receptor–dependent activation of adenylate cyclase (AC) in human embryonic kidney 293 (HEK293) cells (9); and it can couple to β2 adrenergic receptor and receptors for PTH, thyroid-stimulating hormone, and corticotrophin-releasing factor and mediate cAMP generation as efficiently as Gα_s_ in a murine cell line lacking both Gα_s_ and XLα_s_ (10).

**Figure 1:**
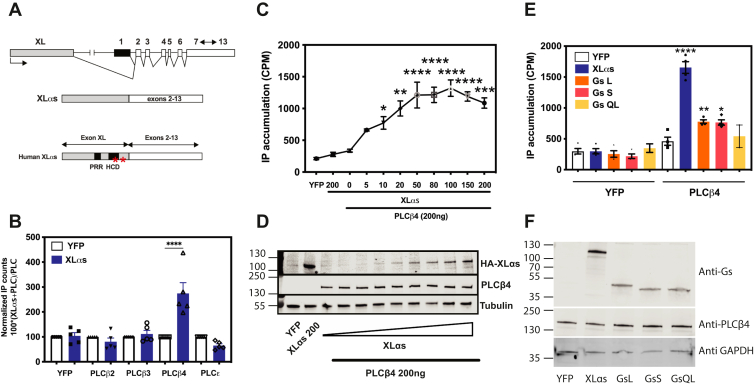
**XLα**_**s**_**activates PLCβ4.***A*, splicing of the XL exon to exons 2 to 13 at the GNAS locus results in XLα_s_. The XL amino-terminal domain contains a proline-rich region (PRR) followed by a highly charged domain (HCD). *Red asterisks* denote two cysteine residues (C287 and C318). *B*, COS-7 cells were transfected with indicated plasmid constructs. About 24 h post-transfection, cells were incubated with F-10 media containing 1.5 mCi/well myo[2-^3^H(N)] inositol and assayed the next day for total [^3^H]inositol phosphate (IP) accumulation, using Dowex AGX8 anion exchange columns as detailed in the Experimental procedures section. ∗∗∗∗ One-way ANOVA test, Bonferroni post hoc test, *p* < 0.0001. *C*, concentration-dependent activation of PLCβ4 by XLα_s_ in COS-7 cells. COS-7 cells were transfected with indicated plasmid constructs. Coexpression of an increasing amount of XLα_s_ (0–200 ng) and a fixed amount of PLCβ4 (200 ng) results in increasing IP accumulation. ∗, ∗∗, ∗∗∗, and ∗∗∗∗ one-way ANOVA, Bonferroni post hoc test, *p* < 0.05, *p* < 0.01, *p* < 0.001, *p* < 0.0001, respectively. *D*, Western blot of XLα_s_ (HA tagged) shows increased XLα_s_ protein expression in corresponding to the amount of XLα_s_ plasmid transfected, whereas PLCβ4 expression is unchanged. *E*, IP accumulation in COS-7 cells transiently transfected with different Gα_s_ variants and PLCβ4. ∗, ∗∗, and ∗∗∗∗ One-way ANOVA, compared with PLCβ4, Bonferroni post hoc test, *p* < 0.05, *p* < 0.01, *p* < 0.0001, respectively. *F*, Western blot of XLα_s_, Gα_s_long, Gα_s_short, and Gα_s_QL shows similar protein expression, whereas PLCβ4 expression is unchanged. Data combined from at least three independent experiments are shown as mean ± SEM. Alpha Stimulating activity polypeptide; GNAS, Guanine Nucelotide binding protein, HA, hemagglutinin; PLCβ4, phospholipase Cβ4; XLα_s_, extra-large stimulatory Gα.

PTH activates Gα_s_ and Gα_q/11_ signaling in renal proximal tubules to regulate serum calcium and phosphate levels through phosphate reabsorption and vitamin D synthesis *in vivo* (11, 12, 13). Surprisingly, XLα_s_ deletion in mice (XLKO) did not significantly affect cAMP production but rather decreased both basal and PTH-stimulated inositol 1,4,5-trisphosphate (IP_3_) production in renal proximal tubules isolated from these mice (14). Expression of XLα_s_ in proximal tubules of XLKO mice rescued basal and PTH-stimulated inositol phosphate (IP) production. Overexpression of XLα_s_ in HEK293 cells enhanced basal and both thrombin- and PTH-stimulated IP production. That changes in IP production occurred in the absence of changes in cAMP in proximal tubules and occurred downstream of thrombin, which does not stimulate cAMP production, argues that XLα_s_-stimulated IP production is not downstream of cAMP. The mechanism for how XLα_s_ enhances IP_3_ production, however, is unknown since no known isoform of phospholipase C (PLC) has been shown to respond to Gα_s_ or XLα_s_.

Generation of IP_3_ involves activation of PLC enzymes, of which five isoforms have been identified to respond to G protein activation (PLCβ1–4 and PLCε). PLC enzymes hydrolyze phosphatidylinositol 4,5-bisphosphate or phosphatidylinositol 4-phosphate (15, 16). Phosphatidylinositol 4,5-bisphosphate hydrolysis generates diacylglycerol and IP_3_. Diacylglycerol regulates the activity of protein kinase C, and IP_3_ mobilizes intracellular Ca^2+^, both of which initiate multiple signaling cascades to regulate a variety of cellular processes (16). All PLCβ isoforms are activated by Gα_q/11_ subtype (17). PLCβ2 and PLCβ3 are also activated by Gβγ subunits (18, 19, 20). PLCε is a downstream effector of virtually every G protein family because of either direct regulation by G protein βγ subunits (21) or *via* indirect activation by small GTPases of the Ras superfamily (16, 22, 23, 24, 25, 26). Significant progress in understanding the biochemical and physiological functions of PLCβ1, PLCβ2, PLCβ3, and PLCε has been made by multiple laboratories including ours. However, much less is known about the PLCβ4 isoform. PLCβ4 is highly homologous to the NorpA PLC protein that mediates the phototransduction cascade in *Drosophila* (27, 28). Known biological functions of PLCβ4 are limited. PLCβ4 knockout mice develop ataxia (29) and have impaired visual processing (30).

In this report, using both cell biology and biochemical approaches, we demonstrate that PLCβ4 is selectively and directly activated by XLα_s_ through a mechanism that differs from canonical effector activation by G protein α subunits. These results likely explain how XLα_s_ regulates phosphatidylinositol (PI) hydrolysis *in vivo* and suggest a mechanism by which Gs-coupled receptors can activate PLC in tissues that express XLα_s_ and PLCβ4.

## Results

### XLα_s_ selectively activates PLCβ4 in transfected COS-7 cells

To begin to understand the mechanistic basis for XLα_s_-dependent regulation of IP production, we screened several PLC isoforms for XLα_s_-dependent activation. COS-7 cells were cotransfected with XLα_s_ and different PLC complementary DNAs (cDNAs), including PLCβ2, PLCβ3, PLCβ4, and PLCε and measured total IP accumulation. This approach has been used extensively to identify upstream regulators of PLC enzymes (23, 31). IP accumulation increased significantly in cells expressing XLα_s_ and PLCβ4 but not in cells that coexpressed XLα_s_ with other PLC isoforms (Fig. 1*B*). These PLC isoforms were all activated by their canonical G protein activators (Fig. S1) in the same assay. Increasing amounts of XLα_s_ cDNA cotransfected with PLCβ4 led to a concentration-dependent increase in IP production (Fig. 1, *C* and *D*).

Because the only difference between XLα_s_ and Gα_s_ is their first exon (Fig. 1*A*), we next investigated whether Gα_s_ can activate PLCβ4, using the similar transfection approach in COS-7 cells. The long and short variants of Gα_s_ resulted in a small but statistically significant increase in IP accumulation (Fig. 1, *E* and *F*). cAMP activates PKA through cAMP generation and Rap through Epac, respectively. However, cotransfection of PKA or Rap with PLCβ4 did not lead to an increase in IP accumulation (Fig. S2), supporting the idea that XLα_s_-dependent cAMP production is not responsible for PLCβ4 activation by XLα_s_.

### XLα_s_ activates PLCβ4 in a reconstituted enzyme assay

**Figure 2:**
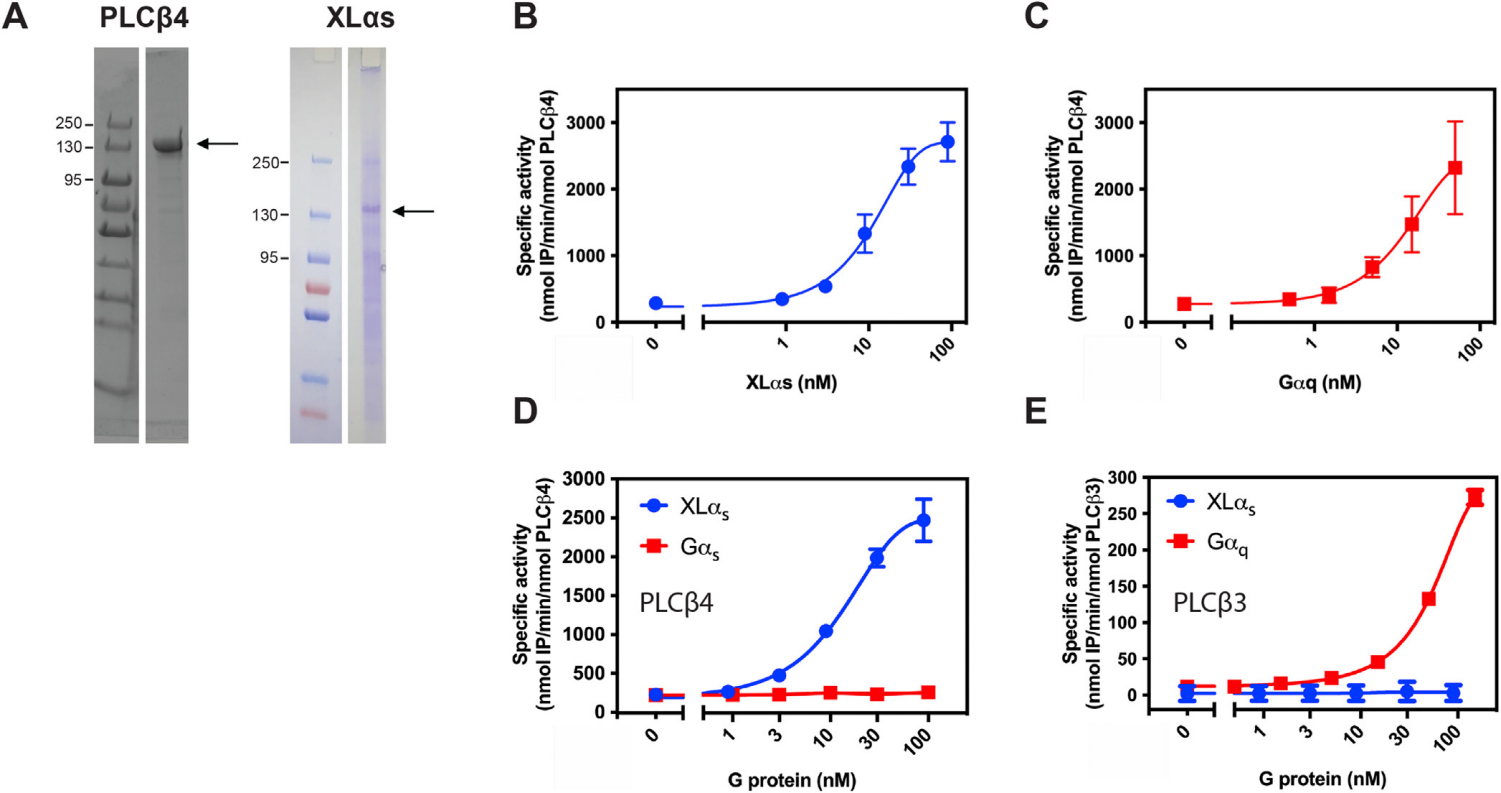
**Direct and specific activation of PLCβ4 by XLα**_**s**_**in a reconstituted enzyme assay.***A*, PLCβ4 and XLα_s_ were purified to ∼95% and ∼30%, respectively. *B*, titration of PLCβ4 activity with XLα_s_ and (*C*) Gα_q_. *D*, recombinant XLα_s_ stimulates PLCβ4 in a concentration-dependent manner, whereas recombinant Gα_s_ does not increase PLCβ4 enzymatic activity. *E*, recombinant Gα_q_ stimulates PLCβ3 in a concentration manner, whereas XLα_s_ does not. Data combined from at least three independent experiments are shown as mean ± SEM. PLCβ4, phospholipase Cβ4; XLα_s_, extra-large stimulatory Gα.

To understand whether the activation of PLCβ4 by XLα_s_ is direct or through other mediators, we partially purified XLα_s_ and PLCβ4 to test the ability of XLα_s_ to activate PLCβ4 *in vitro* with phospholipid vesicles containing PI as the substrate. Through multiple attempts to purify XLα_s_, we achieved a final XLα_s_ preparation at roughly 30% purity with any attempts at further purification leading to protein aggregation (Fig. 2*A*). The XLα_s_ preparation bound to GTPγS although the exact stoichiometry could not be determined because the protein was not pure (Fig. S3). In this reconstituted assay, XLα_s_ increased PLCβ4 enzymatic activity in a concentration-dependent manner (Fig. 2*B*). Direct activation of PLCβ4 by Gα_q_ was tested as a positive control (Fig. 2*C*). Purified Gα_s_ did not activate PLCβ4 (Fig. 2*D*). PLCβ3 was not activated by purified XLα_s_ but was activated by Gα_q_ in the same assay. That the XLα_s_ preparation did not activate PLCβ3 strongly indicates that PLCβ4 activation was not because of contamination of the preparation with Gα_q_ or contamination with a copurifying phospholipase. Overall, these data support the idea that XLα_s_ selectively and directly activates PLCβ4.

### Activation of PLCβ4 by XLα_s_ is independent of activation state and is inhibited by Gβγ

**Figure 3:**
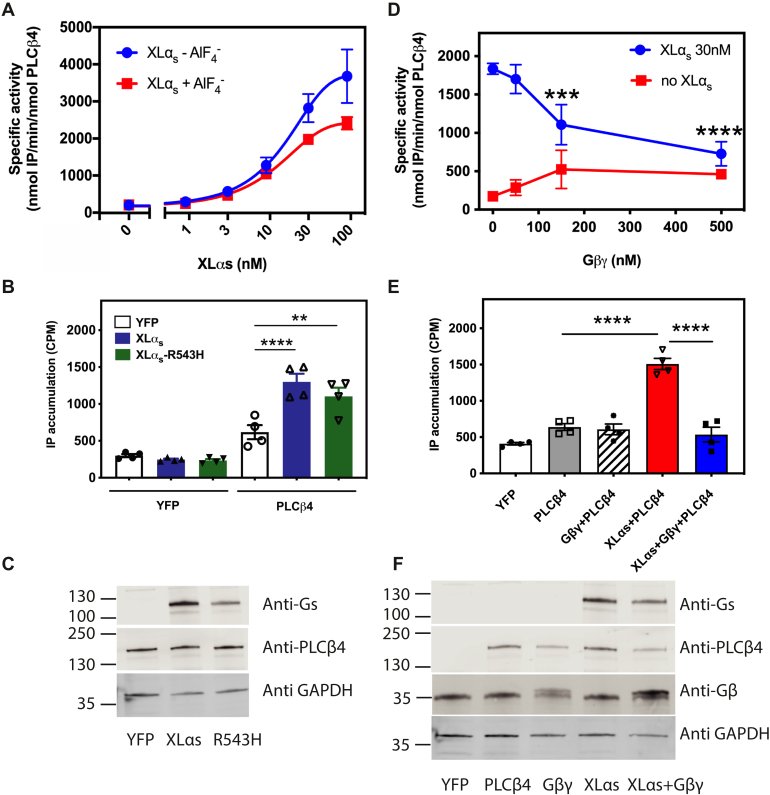
**Activation of PLCβ4 by XLα**_**s**_**is not nucleotide state dependent but is inhibited by Gβγ.***A*, specific activity of PLCβ4 in the presence of different concentrations of XLα_s_ with or without 30 μM AlCl_3_ and 10 mM NaF (AlF_4_^−^). *B*, COS-7 cells cotransfection with PLCβ4 and XLα_s_ or GTPase-deficient XLα_s_ (XLα_s_ R543H) results in higher IP accumulation. XLα_s_ R543H does not lead to a higher IP accumulation. ∗∗ and ∗∗∗∗ One-way ANOVA, Dunnett post test, *p* < 0.01, *p* < 0.0001, respectively. *C*, Western blot of XLα_s_, XLα_s_R543H shows similar protein expression, whereas PLCβ4 expression is unchanged. *D*, effect of addition of purified Gβ_1_γ_2_ on XLα_s_-activated PLCβ4 in reconstituted assay. ∗∗∗ and ∗∗∗∗ Two-way ANOVA, Dunnett post test, *p* < 0.001, *p* < 0.0001, respectively, or in *E*, cellular assay, ∗∗∗∗ one-way ANOVA, Dunnett post test, *p* < 0.0001. *F*, Western blot from COS-7 cells coexpressing PLCβ4 with or without Gβ_1_ and Gγ_2_ as indicated in *E*. Data combined from three to four independent experiments are shown as mean ± SEM. IP, inositol phosphate; PLCβ4, phospholipase Cβ4; XLαs, extra-large stimulatory Gα.

The ability of G protein α subunits to engage and activate their effectors is strongly enhanced in the GTP-bound activated form. Aluminum fluoride (AlF_4_^–^) forms a complex with Gα•GDP, resulting in Gα•GDP•AlF_4_^–^ complex that resembles the activated Gα•GTP. XLα_s_ activated PLCβ4 *in vitro* regardless of whether AlF_4_^–^ was added (Fig. 3*A*). This finding was further confirmed in COS-7 cells coexpressing PLCβ4 with WT XLα_s_ or with XLα_s_ variant (R543H) that is constitutively active with respect to activation of adenylyl cyclase (7). XLα_s_-R543H did not further increase IP accumulation as a result of PLCβ4 activation while having similar protein expression levels as XLα_s_ (Fig. 3, *B* and *C*). However, XLα_s_-mediated PLCβ4 activation *in vitro* was suppressed in a concentration-dependent manner by the addition of purified Gβγ subunits (Fig. 3*D*). Similarly, cotransfection of Gβ_1_γ_2_ also inhibited IP accumulation by XLα_s_-activated PLCβ4 in COS-7 cells (Fig. 3, *E* and *F*).

### Activation of PLCβ4 by XLα_s_ does not require GTP-dependent conformational changes in switch II region

**Figure 4:**
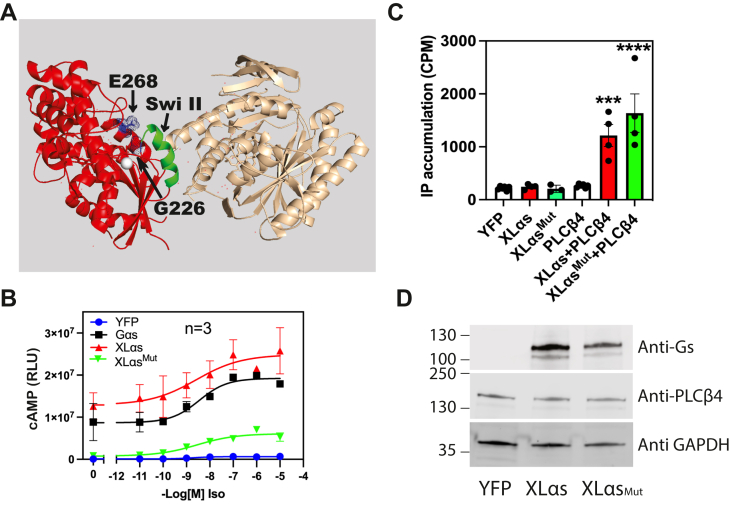
**Loss-of-function mutation in XLα**_**s**_**does not affect its ability to activate PLCβ4.***A*, mutations at glutamate-268 and glycine-226 (*blue*) in Gα_s_ (*red*) render Gα_s_ protein that has impaired agonist-induced cAMP generation. *Green helix* denotes the switch II region that engages adenylyl cyclase (*tan*). *B*, Gα_s_KO HEK293 cells transfected with β2 adrenergic receptor, cAMP Glo, and indicated plasmid constructs (YFP, Gα_s_, XLα_s_, and XLα_s_^Mut^) and cAMP content following isoproterenol addition was assayed as described in the Experimental procedures section. *C*, COS-7 cells were transfected with indicated plasmid constructs, and total IP accumulation was assayed as described for Figure 1*A*. ∗∗∗∗ and ∗∗∗ One-way ANOVA test, Dunnett post hoc test, *p* < 0.0001, *p* < 0.001, respectively. *D*, Western blots show similar protein expression of XLα_s_ and XLα_s_^Mut^, whereas PLCβ4 expression is unchanged. Data combined from three to four independent experiments are shown as mean ± SEM. HEK293, human embryonic kidney 293 cell line; IP, inositol phosphate; PLCβ4, phospholipase Cβ4; XLα_s_, extra-large stimulatory Gα.

Canonical G protein activation upon GTP binding involves a variety of conformational changes that allow for engagement of effectors. In particular, switch II region of Gα_s_ undergoes conformational changes upon GDP–GTP exchange that enhance engagement with AC to mediate its activation. Other G protein α subunits also operate through this mechanism. Loss-of-function mutations of the glycine G226 and glutamate E268 residues in Gα_s_, which interact with residues in switch II region (Fig. 4*A*), are defective in GTP-induced activation of AC (32). We made the analogous double mutant in XLα_s_ (G568A/Q610A; designated as XLα_s_^Mut^). This XLα_s_^Mut^ does not stimulate cAMP production on its own and showed markedly reduced ability to mediate isoproterenol (Iso)-induced cAMP generation compared with the WT XLα_s_ (Fig. 4*B*). However, this mutant activated PLCβ4 to induce IP accumulation similarly to WT XLα_s_ (Fig. 4*C*) and had similar protein expression levels to WT XLα_s_ (Fig. 4*D*). This provides additional support to the idea that XLα_s_ stimulates PLCβ4 in a cAMP-independent manner and shows that XLα_s_ engagement with PLCβ4 likely requires a different structural determinant than switch II region.

### Membrane localization of XLα_s_ is required for full activation of PLCβ4

**Figure 5:**
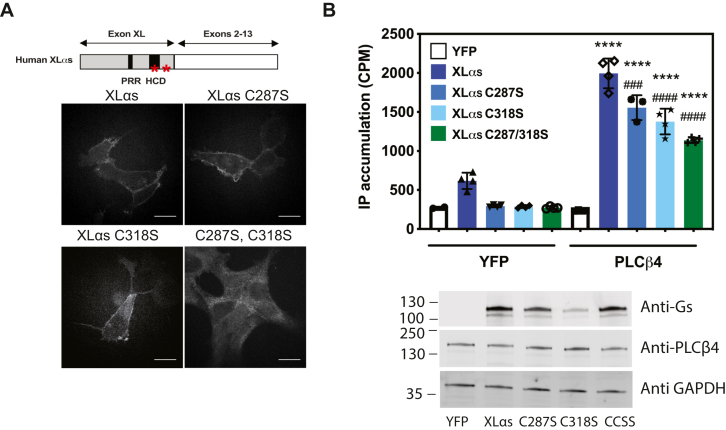
**Plasma membrane localization of XLα**_**s**_**is important for PLCβ4 activation.***A*, schematic diagram of XLα_s_ domain structure, WT XLα_s_ consists of an XL domain, which contains a highly charged domain (HCD), and a proline-rich region (PRRP). *Asterisks* depict the two conserved cysteines in the XL domain. Immunocytochemical analysis of subcellular distribution for WT and Cys-to-Ser mutants of XLα_s_ in HEK293 cells by using an anti-HA antibody. HEK293 cells were transiently transfected with expression constructs encoding HA-tagged WT or Cys-to-Ser mutants of XLα_s_ (Cys-287 and Cys-318). Twenty-four hours after transfection, subcellular localizations of these XLα_s_ mutants were investigated. The scale bar represents 5 μM. *B*, total IP accumulation in COS-7 cells expressing WT XLα_s_ or Cys-to-Ser mutants of XLα_s_ and PLCβ4. ∗∗∗∗*p* < 0.0001 compared with PLCβ4, one-way ANOVA, Tukey post test, ### and #### *p* < 0.001, *p* < 0.0001, respectively, compared with XLα_s_ + PLCβ4, one-way ANOVA, Tukey post test. Western blots show expression of PLCβ4 and different XLα_s_ constructs tested in the IP accumulation assays. Data combined from three to four independent experiments are shown as mean ± SEM. HA, hemagglutinin; HEK293, human embryonic kidney 293 cell line; IP, inositol phosphate; PLCβ4, phospholipase Cβ4; XLαs, extra-large stimulatory Gα.

XLα_s_ tightly localizes to the PM compared with Gα_s_, in part because of the presence of two conserved palmitoylated cysteine residues C287 and C318 and a highly charged domain within the extended N terminus (7). The individual cysteine mutations did not substantially alter PM binding, but mutation of both C287 and C318 to serine significantly decreased the localization of XLα_s_ to the PM (7) (Fig. 5*A*). COS-7 cells were transiently transfected with WT XLα_s_, XLα_s_ (C287S), XLα_s_ (C318S), or XLα_s_ (C287S, C318S) together with PLCβ4 IP accumulation measured. Substitution of either cysteine, or both cysteine residues to serine, showed significantly reduced IP accumulation compared with cells expressing WT XLα_s_ and PLCβ4, although IP accumulation was not entirely abolished (Fig. 5*B*).

### Structure–activity relationship studies of XLα_s_ reveal that targeting Gα_s_ to the PM is sufficient for activation of PLCβ4

**Figure 6:**
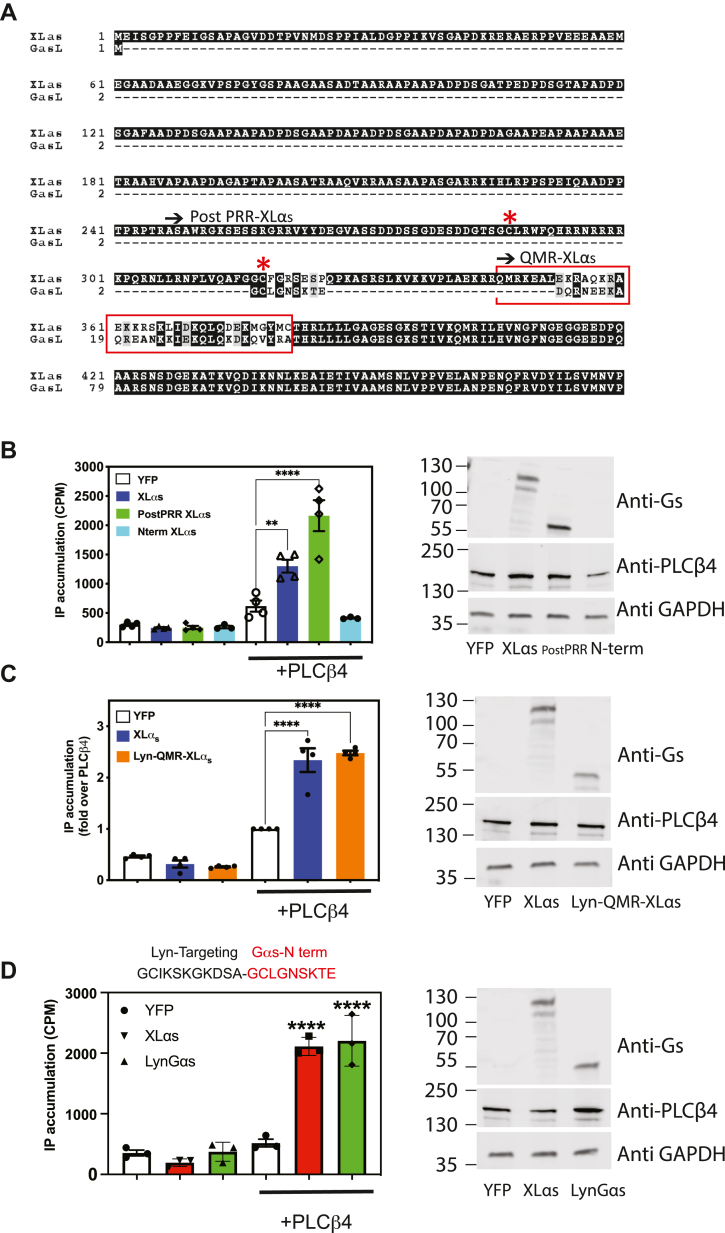
**Identifying the region in XLα**_**s**_**that activates PLCβ4.** Membrane-targeting intact Gα_s_ activates PLCβ4. *A*, sequence alignment of XLα_s_ and the amino terminus of Gα_s_ long (through Gα_s_ amino acid 138. The remainder of Gα_s_ is identical to XLα_s_). *Red asterisks* denote XLα_s_ cysteine 287 and 318. *Arrow* marks the beginning of the post PRR-XLα_s_ and the QMR-XLα_s_ sequence that follow immediately after Lyn membrane targeting motif (Lyn-QMR-XLα_s_). *Red box* denotes the N-terminal α-helical Gβγ interaction domains in XLα_s_ and Gα_s_. *B*–*D*, COS-7 cells were transfected with indicated plasmid constructs, and total IP accumulation was assayed as described for Figure 1*A*; protein expression was examined by SDS-PAGE and immunoblotting. For *D*, above the graph is a schematic depicting the sequence at Lyn-N-terminal Gα_s_ junction in Lyn-Gα_s_. ∗∗ and ∗∗∗∗ One-way ANOVA test, Dunnett post hoc test, *p* < 0.01, *p* < 0.0001, respectively. Data combined from three to four independent experiments are shown as mean ± SEM. IP, inositol phosphate; PLCβ4, phospholipase Cβ4; PRR, prOline-rich region; XLαs, extra-large stimulatory Gα.

Gα_s_ and XLα_s_ are nearly identical except for their N-terminal regions (Figs. 1*A* and 6*A*), yet XLα_s_ activates PLCβ4 with significantly higher efficacy (Fig. 1, *E* and *F*). We created a series of truncation mutations in XLα_s_ and investigated their ability to activate PLCβ4 in cells. A cDNA construct with removal of N-terminal amino acids (amino acids 2–240) beyond the proline-rich region (PRR), and prior to the palmitoylation sites (post-PRR XLα_s_) (7), still markedly increased IP accumulation when cotransfected with PLCβ4 in COS-7 cells (Fig. 6*B*). In addition, a construct that comprises exclusively the N-terminal residues (amino acids 1–381) fused to GFP (Nterm XLα_s_) (33) had no effect on IP accumulation when cotransfected with PLCβ4 (Fig. 6*B*).

We further removed the region extending from the C-terminal end of PRR to the C-terminal end of highly charged domain (amino acids 2–345 removed) and added the Lyn membrane targeting motif (GCIKSKGKDSA) (34) at its N terminus to create Lyn-QMR-XLα_s_. The addition of the Lyn sequence is designed to replace the XLα_s_ membrane targeting determinants lost in this deletion construct and maintain QMR-XLα_s_ association with the membrane. This Lyn-QMR-XLα_s_ construct only differs from the Gα_s_ at its N terminus helix (in *red box*, Fig. 6*A*), which is a putative Gβγ interacting domain in XLα_s_ (7). IP accumulation increased significantly in cells expressing Lyn-QMR-XLα_s_ and PLCβ4 (Fig. 6*C*).

Since QMR-XLα_s_ and Gα_s_ differed by only a short stretch of amino acids corresponding to the amino terminus of Gα_s_, we examined whether addition of the Lyn targeting sequence to the amino terminus of Gα_s_ would enable it to activate PLCβ4. To achieve this, we inserted the Lyn motif at the N terminus of Gα_s_ short (LynGα_s_). Surprisingly LynGα_s_ activated PLCβ4 similarly to XLα_s_ (Fig. 6*D*). This indicates that specific structural features of the unique XLα_s_ N terminus are not required for PLCβ4 activation but rather the ability of XLα_s_ to anchor strongly to the PM allows it to interact with and activate PLCβ4.

### XLα_s_ regulation of PLCβ4 mediates Iso-dependent IP production in osteocytes

**Figure 7:**
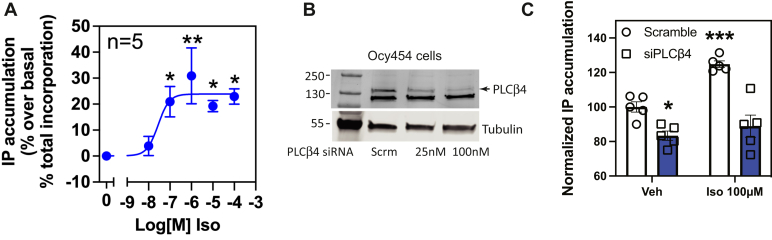
**Isoproterenol-induced IP accumulation in Ocy454 cells is mediated by PLCβ4.***A*, isoproterenol induces a concentration-dependent increase in IP accumulation in osteocyte-like Ocy454 cells. ∗*p* < 0.05 and ∗∗*p* < 0.01, one-way ANOVA, Dunnett post test. *B*, representative immunoblot showing reduced PLCβ4 expression in Ocy454 cells transiently transfected with PLCβ4 siRNA or control scramble siRNA (SmartPool) (Scrm, scrambled). *C*, IP1 concentrations were significantly diminished at baseline and after isoproterenol stimulation in PLCβ4 knockdown-Ocy454 cells. ∗ and ∗∗∗ Two-way ANOVA test, Sidak post hoc test, *p* < 0.05, *p* < 0.001, respectively. Data combined from five independent experiments are shown as mean ± SEM. IP, inositol phosphate; PLCβ4, phospholipase Cβ4.

It has been shown that ablation of XLα_s_ in isolated proximal tubule–enriched renal cortices and osteocyte-like Ocy454 cell line represses IP_3_ generation (14, 35). Because PLCβ4 is activated by XLα_s_, we examined if PLCβ4 mediates the effect of XLα_s_ in maintaining basal and Iso-dependent regulation of IP production in Ocy454 cells. Iso stimulation of Ocy454 cells resulted in a small but statistically significant increase in IP production (Fig. 7*A*). Transfection of these cells with a pool of PLCβ4-directed siRNA oligonucleotides depleted PLCβ4 protein (Fig. 7*B*), whereas control (scrambled) oligonucleotides did not. Ocy454 cells with depleted PLCβ4 had reduced basal and Iso-stimulated IP production compared with cells transfected with scrambled siRNA oligonucleotides. This finding supports a role for PLCβ4 in XLα_s_-dependent regulation of IP signaling in osteocytes.

## Discussion

In this study, we present evidence that PLCβ4 is a direct effector of an amino terminally extended variant of Gα_s_, XLα_s_. This regulation is specific to PLCβ4 relative to other PLC isoforms, revealing a novel potential mechanism for stimulation of IP production downstream of Gs-coupled receptors that is independent of cAMP. XLα_s_ and Gα_s_ are both encoded by the GNAS locus. Early reports demonstrated that XLα_s_ had similar properties to Gα_s_ in its ability to bind and dissociate from Gβγ subunits and to regulate AC (4, 8). Initially, results also suggested that XLα_s_ could not couple to adrenergic receptors in reconstituted S49cyc-membranes lacking Gα_s_ (8). However, subsequent studies demonstrated that XLα_s_ and Gα_s_ have similar functions in mediating receptor-dependent stimulation of cAMP production *via* adenylyl cyclase activation (9, 10).

We demonstrate here that unlike Gα_s_, XLα_s_ activates PLCβ4, which is considered to be a canonical Gα_q/11_ effector. Since both Gα_s_ and XLα_s_ stimulate cAMP production, this indicates that the effect of XLα_s_ on IP production is not because of the actions of the cAMP targets PKA and Epac in cells. Several other lines of data support a cAMP-independent mechanism including a lack of effect of PKA transfection or Epac inhibition on PLCβ4 activation (Fig. S2). Compellingly, a mutation that disables the active conformation of the switch 2 helix in XLα_s_ (Fig. 4) abolishes its ability to stimulate cAMP production in the absence of receptor activation but does not alter its ability to support activation of PLCβ4.

Our biochemical reconstitution experiments also support a mechanism where XLα_s_ directly activates PLCβ4 independent of cAMP. This approach as well as Cos cell cotransfection experiments have established the canonically accepted mechanisms for regulation of PLCβ isoforms by G protein subunits (20, 31, 36, 37, 38, 39). A caveat is that the preparation of XLα_s_ is impure and leaves the possibility that a contaminating component of the preparation is activating or facilitating activation of PLCβ4 or has intrinsic PLC activity. Multiple controls strongly argue against contamination by other G proteins or PLCs including the inability of the preparation to activate PLCβ3, which is activated by both Gα_q_ and Gβγ. In addition, the majority of the biochemical properties of XLα_s_ in the *in vitro* PLC assays were recapitulated in the intact cell cotransfection assay including nucleotide-independent activation.

We observed a statistically significant small activation of PLCβ4 by Gα_s_ in cells that was not observed in the *in vitro* reconstitution experiments. One possibility is that in cells there are additional regulatory mechanisms downstream of Gα_s_ and cAMP that can alter PLCβ4 activation independently of direct activation of PLCβ4 by XLα_s_. An alternate possibility is that *in vitro* Gα_s_ does not interact with the phophatidylethanolamine:PI vesicle bilayer that supplies the PI substrate and supports G protein–PLC interactions, whereas XLα_s_ is able to bind to this membrane surface allowing it to engage with PLCβ4.

Selective knockout of XLα_s_ in mice decreased basal and PTH-dependent IP production in renal proximal tubules but, surprisingly, did not result in a decrease in cAMP production (14). IP production was enhanced in kidney proximal tubules isolated from mice with transgenic expression of XLα_s_ and in HEK293 cells transfected with XLα_s_. PTH stimulates urinary phosphate excretion, which is known to be regulated, at least partly, by IP_3_ production. Serum phosphate levels were significantly increased in XLα_s_ knockout mice, which could be attributed to a resistance to PTH-stimulated IP_3_ production. Heretofore, no known mechanisms for PLC regulation could explain these results. Regulation of PLCβ4 by XLα_s_ provides a likely mechanistic basis for these observations in mice and other systems. PLCβ4 is expressed in the proximal convoluted tubule (40) as well as Ocy454 osteocyte–like cells (Fig. 7*B*), in which XLα_s_ is also expressed and mediates IP_3_ production.

The mechanism for XLα_s_-dependent regulation of PLCβ4 diverges from classical mechanisms for G protein–dependent effector activation. In the biochemical reconstitution experiments, activation was independent of nucleotide status and in cell transfection studies did not rely on the switch II region classically involved in effector engagement. This property has been observed in Gα_s_-dependent activation of adenylyl cyclase, where purified Gα_s_ activated adenylyl cyclase in both GDP and GTP-bound states, albeit with different potencies (41). The small G protein K-ras has also been reported to interact with its effector argonaute 2 independently of its nucleotide state (42). Despite the nucleotide-independent activation of PLCβ4 by XLα_s_, addition of Gβγ subunits inhibited the actions of XLα_s_ on PLCβ4 both in cells and with purified proteins. This suggests a mechanism whereby receptors could regulate XLα_s_–PLCβ4 interactions based on receptor-dependent dissociation of XLα_s_ from Gβγ subunits.

Our structure function analysis demonstrated that a primary determinant of XLα_s_-dependent of PLCβ4 is its unique mode of membrane targeting relative to Gα_s_. The Gα_s_ domain at the carboxy terminus of XLα_s_ is identical to Gα_s_ (Figs. 1*A* and 6*A*). Surprisingly, both XLα_s_ and a Gα_s_ variant containing a Lyn-targeting sequence at the amino terminus activate PLCβ4 to similar extents. The Lyn PM targeting sequence Gly Cys Ile Lys Ser Lys Gly Lys Asp Ser Ala is myristoylated at Gly1 and palmitoylated at Cys2 and is enriched in positively charged amino acids that all contribute to specific PM localization (34, 43). The XLα_s_ amino terminus is also enriched in positively charged amino acids. Gα_s_ is palmitoylated at its amino terminus at Cys3 and is not enriched in positively charged amino acids (44). Gα_s_ dissociates from the PM upon activation, whereas XLα_s_ does not (7, 45). How Gα_s_ is anchored to the membrane may modulate its orientation at the membrane relative to its targets, with the unique amino terminus of XLα_s_ orienting the Gα_s_ domain such that it can engage and activate PLC. Alternatively, prolonged residency of XLα_s_ at the PM allows for engagement with PLCβ4. The precise molecular mechanisms for how XLα_s_ binds and activates PLCβ4, however, requires further study.

## Experimental procedures

### Plasmid constructs and cloning

The Gateway entry vector encoding PLCβ4 was purchased from Genecopoeia (catalog no.: GC-Y5168-CF-GS). QuikChange mutagenesis was performed to add stop codon to the ORF. Gateway pDEST10 vector was purchased from Thermo Fisher Scientific (catalog no.: 11806015). Destination vector pEZYegfp was a gift from Yu-Zhu Zhang (Addgene; plasmid #18671). The complete PLCβ4 ORF was transferred from the entry vector to pEZYegfp or Gateway pDEST10 vectors using Gateway LR Clonase II Enzyme Mix (Invitrogen; catalog no.: 11791-020), following the manufacturer’s protocol, resulting in a mammalian expression vector encoding N-terminally tagged enhanced GFP PLCβ4 and a baculovirus vector encoding N-terminally tagged 6xHis PLCβ4. XLα_s_ in pcDNA3.1 was previously described. N-terminal-hexahistidine-tagged XLα_s_ (His_6_-XLα_s_) was synthesized by inserting sequences encoding six histidine residues after the start codon methionine of pFastBac-XLα_s_ made in house. Truncation mutagenesis was done using Q5 Site-Directed Mutagenesis kit (NEB). The Lyn N-terminal sequence (Gly Cys Ile Lys Ser Lys Gly Lys Asp Ser Ala—GCIKSKGKDSA) (34) was inserted at the N terminus of Gα_s_ right after the start codon to create Lyn-Gα_s_ construct. Lyn-QMR-XLα_s_ was created by performing deletion of residues 2 to 345 in XLα_s_ and then inserted the Lyn sequence between the start codon Met and Glu346.

### Protein purification

SF9 and High Five insect cells were maintained in Sf-900 II serum-free media. Bacmids and baculoviruses were made following the Bac-to-Bac baculovirus expression system protocol (Thermo Fisher Scientific).

Purification of 6xHis PLCβ4 followed previously described protocols (46). Briefly, High Five insect cells were infected with baculovirus at a density between 1.5 × 10^6^ and 2 × 10^6^ cells/ml at a multiplicity of infection of 1. After 48 h, cells were harvested by centrifugation, snap frozen in liquid nitrogen, and stored at −80 °C. Frozen insect cell pellets expressing His6 PLCβ4 were lysed in 15 ml lysis buffer (per liter of insect cell culture) containing 20 mM Hepes, pH 8, 50 mM NaCl, 10 mM β-mercaptoethanol (β-ME), 0.1 mM EDTA, 0.1 M EGTA, 0.1 mM DTT, protease inhibitors including 133 μM PMSF, 21 μg/ml tosyl-l-lysine chloromethyl ketone and tosyl-l-phenylalanine chloromethyl ketone, 0.5 μg/ml aprotonin, 0.2 μg/ml leupeptin, 1 μg/ml pepstatin A, 42 μg/ml tosyl-L-arginine methyl ether, 10 μg/ml soybean trypsin inhibitor by subjecting the cell suspension to four cycles of thawing in a 37 °C water bath and snap freezing in liquid nitrogen. The lysate was diluted with 45 ml cold lysis buffer with addition of NaCl to a final concentration of 1 M and centrifuged at 40,000 rpm using a Ti60 rotor. The supernatant was collected and diluted 5× with buffer containing 10 mM Hepes, pH 8, 10 mM β-ME, 0.1 mM EDTA, 0.1 M EGTA, 0.5% polyoxyethylene lauryl ether (C_12_E_10_), and protease inhibitors. The diluted supernatant was then centrifuged at 100,000*g*, and the supernatant was loaded onto a nickel–nitrilotriacetic acid column pre-equilibrated with buffer A (20 mM Hepes, pH 8, 100 mM NaCl, 10 mM β-ME, 0.1 mM EDTA, and 0.1 M EGTA). The column was washed with three column volumes (CVs) of buffer A, followed by three CVs of buffer A supplemented with 300 mM NaCl and 10 mM imidazole. The protein was eluted from the column with 3 to 10 CVs of buffer A, supplemented with 200 mM imidazole. Proteins were concentrated and loaded onto a gel filtration Superdex column equilibrated with buffer containing 20 mM Hepes, pH 8, 200 mM NaCl, 2 mM DTT, 0.1 mM EGTA, and 0.1 mM EDTA. Fractions of His6 PLCβ4 at greater than 95% purity were confirmed by SDS-PAGE and Coomassie staining, pooled, concentrated, and snap frozen in liquid nitrogen. Protein concentrations were determined by Nanodrop absorbance at 280 nm and confirmed by a bicinchoninic acid protein assay.

6xHis-XLα_s_ was coexpressed with Gβ_1_γ_2_ in High Five cells and purified using a nickel–nitrilotriacetic acid affinity column. Briefly, High Five cells were harvested 48 h postinfection. Cell pellets were suspended in 15 ml lysis buffer (20 mM Hepes, pH 8.0, 100 mM NaCl, 1 mM EDTA, 2 mM MgCl_2_, 10 mM β-ME, 10 μM GDP, and protease inhibitors) and subjected to four freeze–thaw cycles with liquid nitrogen to promote cell lysis. The resulting lysate was further diluted with lysis buffer to 80 ml and centrifuged at 35,000 rpm for 1 h. Ensuing membrane pellets were resuspended in extraction buffer (20 mM Hepes, pH 8.0, 100 mM NaCl, 1 mM MgCl_2_, 10 mM β-ME, 10 μM GDP, and protease inhibitors) and homogenized. Membrane proteins were extracted by adding sodium cholate to a final concentration of 1% (v/v) and isolated *via* centrifugation at 35,000 rpm for 45 min. The resulting supernatant was diluted 1:5 with Ni^2+^ loading buffer A (20 mM Hepes, pH 8.0, 100 mM NaCl, 0.1 mM MgCl_2_, 5 mM β-ME, 40 mM imidazole, 10 μM GDP, 0.5% C_12_E_10_, and protease inhibitors) and loaded onto a 1 ml HisTrap HP column (Cytiva) at 0.5 ml/min. After washing with 25 ml of Ni^2+^ load buffer A to remove nonspecific impurities, the HisTrap column was warmed to room temperature and subjected to five 2 ml washes with aluminum fluoride elution buffer (20 mM Hepes, pH 8.0, 300 mM NaCl, 10 mM MgCl_2_, 5 mM β-ME, 30 mM imidazole, 10 μM GDP, 0.5% C_12_E_10_, protease inhibitors, 10 mM NaF, and 30 μM AlCl_3_) to elute Gβ_1_γ_2_. The column was then returned to 4 °C and equilibrated with 5 ml of FPLC buffer A (20 mM Hepes, pH 8.0, 100 mM NaCl, 1.25 mM MgCl_2_, 5 mM β-ME, 60 mM imidazole, 10 μM GDP, 1% CHAPS, and protease inhibitors). 6xHis-XLα_s_ was eluted with a linear imidazole gradient constructed from 60 to 500 mM imidazole. Fractions of 1 ml were collected and analyzed using SDS-PAGE on 4 to 20% Tris–glycine Mini-Protean gels (Bio-Rad) followed by Coomassie staining. Fractions containing significant 6xHis-XLα_s_ (molecular weight ∼111 kDa) identified by SDS-PAGE, Coomassie blue staining, and Western blotting were pooled and concentrated, flash-frozen in liquid nitrogen, and stored at −80 °C until use for activity assay.

### Cell culture and [^3^H]-IPx accumulation assay

COS-7 cells were maintained in Dulbecco's modified Eagle's medium (DMEM) supplemented with 10% fetal bovine serum (FBS) and 1% penicillin/streptomycin (pen/strep) at 37 °C with 5% CO_2_. Reverse transfection using Lipofectamine 2000 (Thermo Fisher Scientific) was adapted from manufacturer’s protocol. A maximal amount of DNA/well/24-well plate was 450 ng at a DNA:lipofectamine 2000 ratio of 1:3. COS-7 cells in antibiotics-free DMEM supplemented with 10% FBS were mixed with the DNA:lipofectamine 2000 in a 24-well plate at 100,000 cells/well. Approximately 24 h after transfection, the media was replaced with Ham’s F10 media supplemented with 1.5 mCi/well myo[2-^3^H(N)] inositol (PerkinElmer) and incubated overnight. 10 mM lithium chloride was then added to the cells and incubated for one hour to inhibit the activity of inositol phosphatases. If using agonist, agonist was added immediately after lithium chloride. Media was aspirated, and cells were washed once with ice-cold PBS, followed by the addition of 300 μl ice-cold 50 mM formic acid/well for 1 h for extraction of [^3^H]-IPs. Extracts were transferred to Dowex AGX8 anion exchange columns in a 96-well vacuum manifold to isolate the IPs. Columns were washed six times with 50 mM formic acid, three times with 100 mM formic acid, and then the IPs were eluted with buffer containing 1.2 M ammonium formate and 0.1 M formic acid into a 96-well plate. The eluates were transferred to scintillation vials, and 4 ml of EcoLume Scintillation Cocktail (MP Biomedicals) was added to each vial and counted. All experiments were performed at least three times in triplicate.

Ocy454 cells (47) were maintained in minimum essential media (MEM) supplemented with 10% FBS and 1% pen/strep at 33 °C with 5% CO_2_. Before plating for transfection, Ocy454 cells were cultured at 37 °C for 5 days to differentiate into osteocytes. Cells then were plated in 96-well plates at 30,000 cells/well on day 6 and maintained in MEM supplemented with 10% FBS and 1% pen/strep at 37 °C with 5% CO_2_. For PLCβ4 knockdown, ON-TARGETplus SmartPool scrambled or mouse PLCβ4 (18798) siRNA (Dharmacon-PerkinElmer) was mixed with DharmaFECT reagent in OptiMEM media, and cells were transfected with 100 nM final concentration of siRNA. IPs were extracted and analyzed as described previously.

### PI hydrolysis IP-One homogeneous time-resolved fluorescence assay

PI hydrolysis was measured using a modified version of the commercially available IP-One assay (IP-One G_q_ Kit; Cisbio). Assay of PLCβ activity has been described previously except conditions were modified to use PI as the substrate for compatibility with the IP-One Assay kit (48). Hen egg white phosphatidylethanolamine and soy PI (Avanti Polar Lipids) were mixed and dried under nitrogen. Lipids were resuspended in sonication buffer (50 mM Hepes, pH 7.0, 80 mM KCl, 3 mM EGTA, and 1 mM DTT) and sonicated giving a final concentration of 300 μM phophatidylethanolamine and 750 μM PI. Assays contained 50 mM Hepes, pH 7, 80 mM KCl, 16.67 mM NaCl, 0.83 mM MgCl, 3 mM DTT, 1 mg/ml bovine serum albumin (BSA), 2.26 mM Ca^2+^, and varying amounts of PLCβ4 variant proteins and/or G proteins. XLα_s_ activity was also tested for intrinsic PI hydrolysis activity in the protein preparation. Protein concentrations are indicated in the figure legends. Control reactions contained the same components but lacked CaCl_2_. Reactions were initiated by addition of liposomes and transferred to 37 °C for 5 min. Reactions were quenched upon addition of 5 μl quench buffer (100 mM Hepes, pH 7, 160 mM KCl, 1 mM DTT, and 210 mM EGTA), and 14 μl of each reaction was then transferred to a 384-well plate (Greiner Bio-One). For IP detection, D2-labeled IP1 (fluorescence acceptor) and anti-IP1 cryptate (fluorescence donor) were preincubated with Detection Buffer (Cisbio). 3 μl of D2-labeled IP1 and 3 μl anti-IP1 cryptate were then added to each well used in the 384-well plate. Positive assay controls contained 50 mM Hepes, pH 7, 80 mM KCl, 16.67 mM NaCl, 0.83 mM MgCl, 3 mM DTT, 1 mg/ml BSA, 2.26 mM Ca^2+^, D2-labeled IP1, and anti-IP1 cryptate, whereas negative assay controls contained all components except D2-labeled IP1. The plate was then incubated for one hour in the dark at room temperature, followed by centrifugation at 1000*g* for 1 min. Plates were read with a Varioskan LUX Multimode plate reader (Thermo Fisher Scientific) at 610 and 665 nm. IP1 was quantified using a standard curve and data reduction protocol for normalization (Cisbio). Data were plotted, and statistics were performed using GraphPad Prism 7.0a (GraphPad Software, Inc).

### SDS-PAGE and immunoblotting

Gel electrophoresis and Western blotting were performed as previously described (46). In brief, after transfer, the membrane was incubated with Tris-buffered saline buffer supplemented with 0.1% Tween-20 (TBS-T) and 5% BSA at room temperature for 30 min on a shaker, then probed with primary antibodies (anti-PLCβ4 [Sigma–Aldrich; catalog no.: HPA007951], anti-Gαs [Sigma–Aldrich; catalog no.: 06-237], anti-Gβ [in house], anti-GAPDH [Invitrogen; catalog no.: MA5-15738]) diluted 1:1000 in TBS buffer supplemented with 0.1% Tween-20 and 3% BSA at 4 °C overnight. The membrane was washed with TBS-T four times and probed with secondary antibody (goat anti-rabbit immunoglobulin G, DyLight 800 (Invitrogen; catalog no.: SA535571) at room temperature for 1 h. After another four washes with TBS-T, immunoreactive proteins were visualized using Li-Cor Odyssey CLx and analyzed using Image Studio Lite software (Li-Cor).

### Immunocytochemistry

Cells were grown and transfected in 8-well chamber slides with cover (Nunc Lab-TekII; catalog no.: 154534). Cells were washed three times with PBS and fixed with 4% paraformaldehyde in PBS for 20 min. Cells were permeabilized and blocked with 0.1% saponin and 0.5% BSA in PBS for 1 h. Cells were incubated with a rabbit antihemagglutinin antibody (Abcam; catalog no.: ab137838) and then incubated with Alexa Flour 568-conjugated anti-rabbit immunoglobulin G (Invitrogen; catalog no.: A11036). The immunoreactivity was visualized and analyzed by using a spinning disc confocal fluorescent microscope at 100×.

### Quantification of cAMP generation

pGloSensor-22F cAMP Plasmid construct (Promega) was a gift from Dr Manojkumar Puthenveedu (University of Michigan). The GNAS knockout HEK293T cell line as gift from Kirill Martemyanov (University of Florida, Scripps) (49) was maintained in DMEM supplemented with 10% FBS, glutamine, 100 U/ml penicillin, 100 μg/ml streptomycin in an incubator at 37 °C in an atmosphere of 5% CO_2_, and 95% O_2_. Cells were transiently cotransfected with PTH1R, pGloSensor-22F, and different Gα_s_, XLα_s_, or chimera plasmid constructs in tissue culture–treated solid white 96-well plate (Costar; catalog no.: 3917). cAMP assays were performed 24 h post-transfection. Cells were equilibrated with Leibovitz’s media (Gibco) containing 150 μg/ml d-luciferin potassium salt (GoldBio) for 1 h in 37 °C incubator. After equilibration, luminescence was read before and after treating cells with varying concentration of Iso (MP Biomedicals; catalog no.: 151368) Iso using Varioskan LUX Multimode Microplate Reader (Thermo Fisher Scientific).

## Data availability

All data are included in the article, but primary data files are available upon request to Alan Smrcka.

## Supporting information

This article contains supporting information.

## Conflict of interest

The authors declare that they have no conflicts of interest with the contents of this article.
