# Supplementary material for: A naturally occurring membrane-anchored Gαs variant, XLαs, activates phospholipase Cβ4
Source: J Biol Chem. 2022 Jun 13;298(8):102134. doi: 10.1016/j.jbc.2022.102134 (PMC9294334; doi:10.1016/j.jbc.2022.102134)
Supplement: Supporting information [file mmc1.docx]

**Supporting information**

**
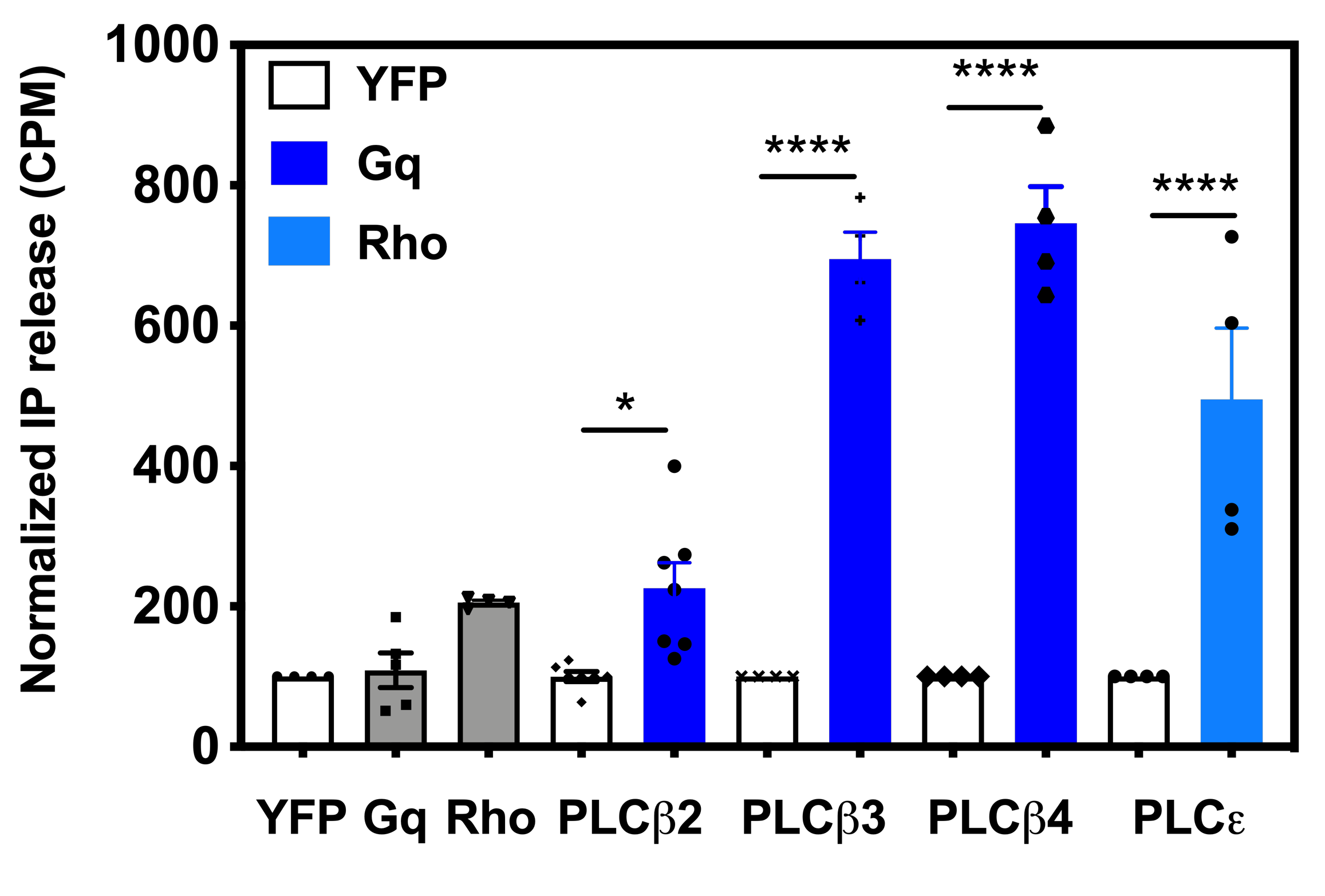
**

**Figure S1.** Co-expression of PLCβ and ε with their canonical activators (Gα_q_ and Rho, respectively) in COS-7 cells shows that Gα_q_ activates PLCβ2, 3 and 4; and Rho activates PLCε, measured by an increase in IP accumulation. ****, * one-way ANOVA test, Bonferroni post-hoc test, p < 0.0001, p < 0.05, respectively.

**
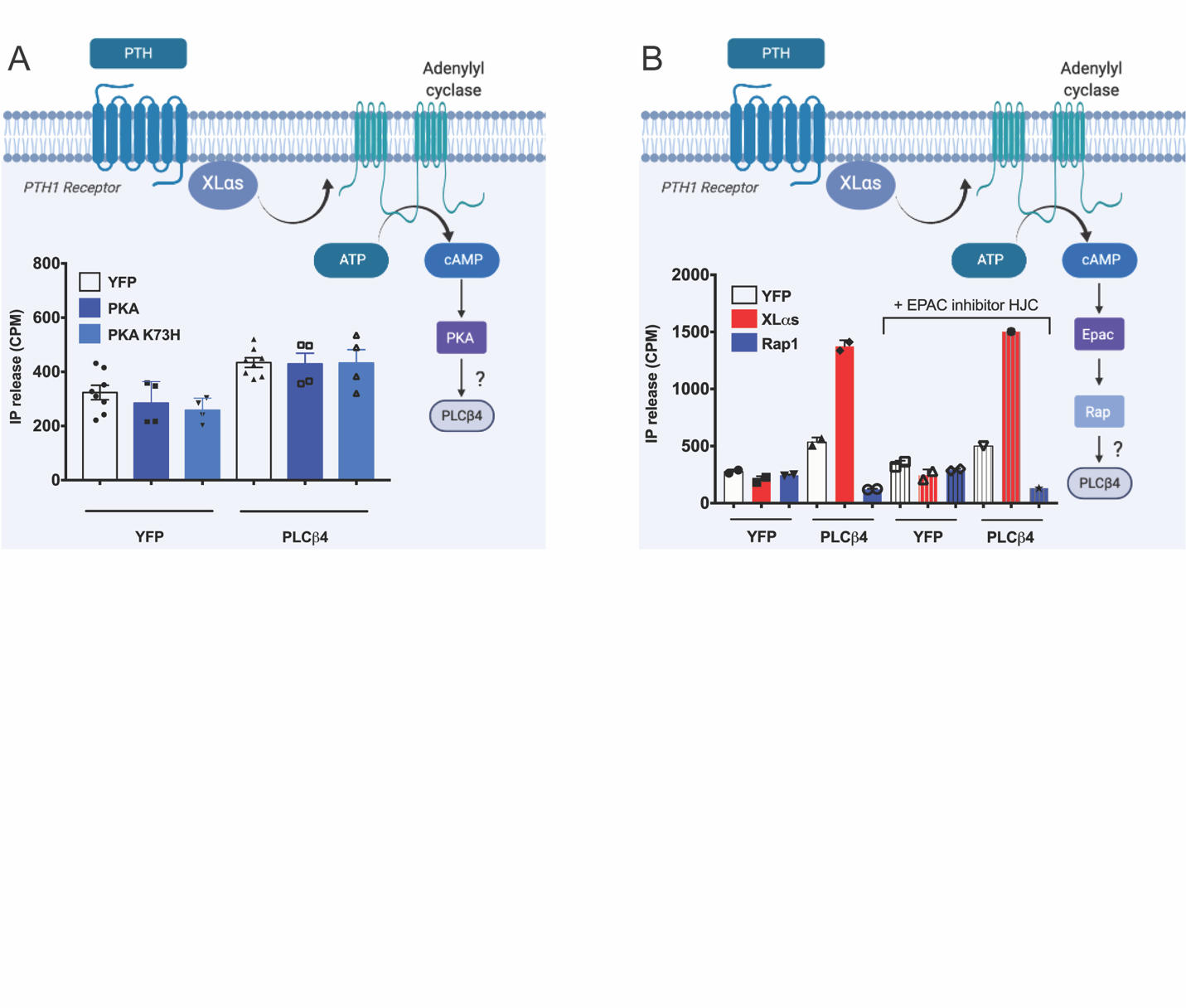
Figure S2.** XLα_s_ does not activate PLCβ4 through cAMP-mediated mechanisms. COS-7 cells were transfected with indicated plasmid constructs and IP accumulation was measured. **A.** Co-transfection of COS-7 cells with PLCβ4 and PKA does not alter IP accumulation. **B.** COS-7 cells were transfected with XLα_s_ and PLCβ4 and treated with or without Epac-selective inhibitor HJC0726. The Epac inhibitor does not alter XLα_s_-stimulated IP accumulation. Rap1 does not activate PLCβ4.

**
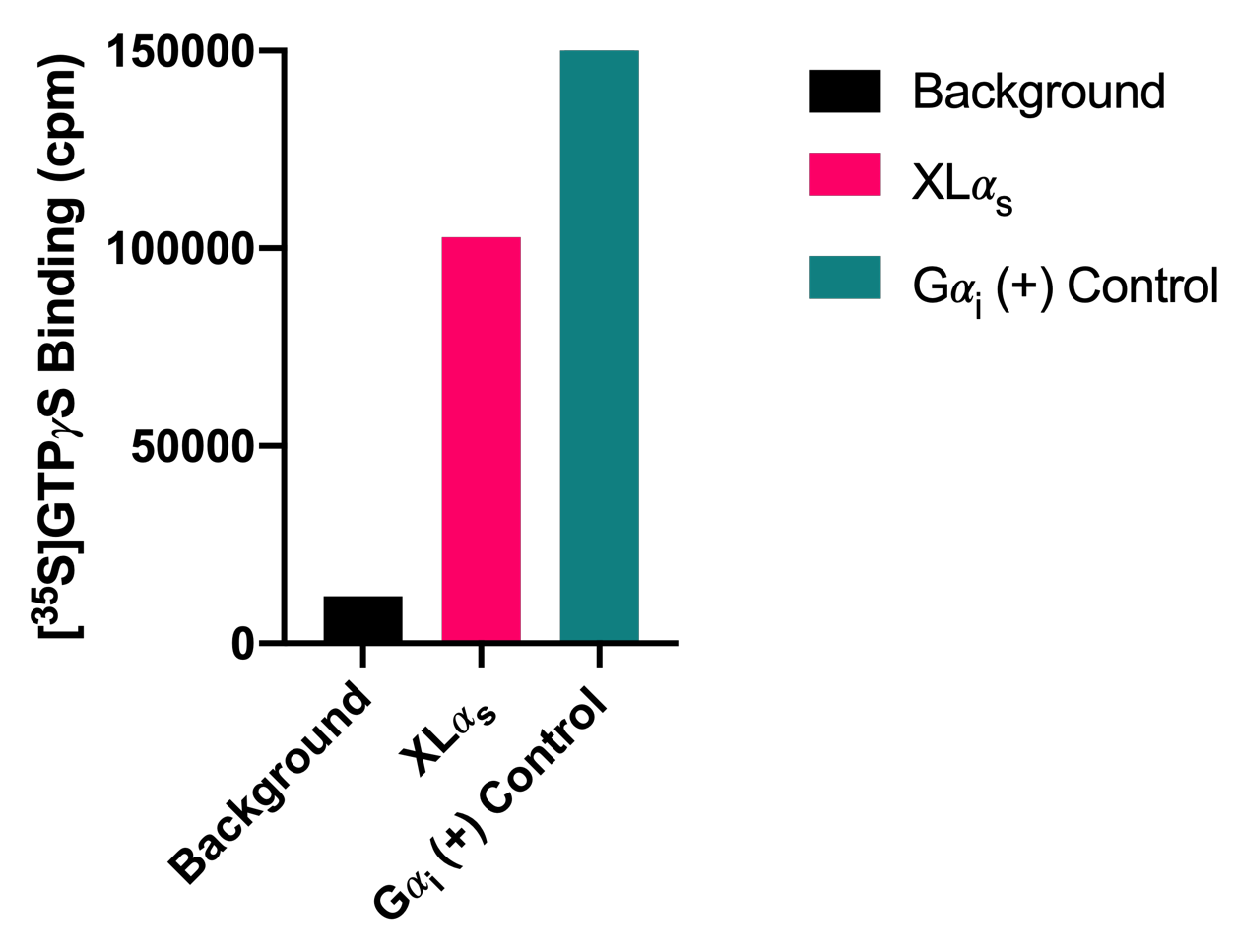
Figure S3. XLα_s_ and Gα_i_ purified from High5 insect cells are active in GTPγS assay.** Purified Gα subunits (XLα_s_ or Gα_i_) were diluted to 1 𝜇M in pre-incubation buffer [50 mM HEPES pH 8.0, 1 mM DTT, 1 mM EDTA, 0.1 % (v/v) C_12_E_10_] and treated with [^35^S]GTPγS binding buffer (50 mM HEPES, pH 8.0, 1 mM DTT, 1 mM EDTA, 40 mM MgCl_2_, 200 mM NaCl, 2 μM unlabeled GTPγS, and ~300,000 cpm [^35^S]GTPγS) for 1 hour at 30 ℃. Reaction contents were poured onto Protran BA85 nitrocellulose filters (Cytiva) and washed with a chilled solution containing 20 mM Tris pH 8.0, 25 mM MgCl_2_, and 100 mM NaCl. Filters were then dried and subjected to liquid scintillation counting.
